# MS^2^PIP prediction server: compute and visualize MS^2^ peak intensity predictions for CID and HCD fragmentation

**DOI:** 10.1093/nar/gkv542

**Published:** 2015-05-18

**Authors:** Sven Degroeve, Davy Maddelein, Lennart Martens

**Affiliations:** 1 Department of Medical Protein Research, VIB, A. Baertsoenkaai 3, B-9000 Ghent, Belgium; 2 Department of Biochemistry, Faculty of Medicine and Health Sciences, Ghent University, A. Baertsoenkaai 3, B-9000 Ghent, Belgium

## Abstract

We present an MS^2^ peak intensity prediction server that computes MS^2^ charge 2+ and 3+ spectra from peptide sequences for the most common fragment ions. The server integrates the Unimod public domain post-translational modification database for modified peptides. The prediction model is an improvement of the previously published MS^2^PIP model for Orbitrap-LTQ CID spectra. Predicted MS^2^ spectra can be downloaded as a spectrum file and can be visualized in the browser for comparisons with observations. In addition, we added prediction models for HCD fragmentation (Q-Exactive Orbitrap) and show that these models compute accurate intensity predictions on par with CID performance. We also show that training prediction models for CID and HCD separately improves the accuracy for each fragmentation method. The MS^2^PIP prediction server is accessible from http://iomics.ugent.be/ms2pip.

## INTRODUCTION

Tandem mass spectrometry (MS^2^) provides the means to match the signals observed by a mass spectrometer with the chemical entities that generated them. This technology provides for a wide range of applications, one of them being the identification and quantification of the proteins in a sample in the form of digested peptides.

MS^2^ produces signal (peaks) spectra that contain information about the chemical dissociation pattern of a peptide that was forced to fragment using methods such as collision induced dissociation (1) (CID) and higher-energy collisional dissociation (2) (HCD). The signal peaks in an MS^2^ spectrum indicate the presence of a peptide fragment ion with a specific mass. The intensity of a peak at a certain mass is dependent on many factors such as the efficiency of the cleavage that generated the fragment and the proteotypicity of the fragment ion.

MS^2^PIP (3) is a model for predicting the MS^2^ peak intensities for the most common types of fragment ions (b+, y+, b++ and y++) from a peptide sequence. MS^2^PIP was trained using machine learning from examples of known peptide-to-spectrum matches (PSMs), where the spectra were generated by an Oribtrap-LTQ mass spectrometer. Since the publication of MS^2^PIP we have made some improvements to the prediction model that allows this web server to predict MS^2^ peak intensities for all peptides, not just those with lengths within [8,28] as in the original version. In addition, we also trained and added a prediction model for HCD fragmentation based on the new model. We show that this HCD model also computes accurate peak intensity predictions. The new prediction models do however, require a large memory footprint (>16 Gb) for predicting the peak intensities.

We therefore built the MS^2^PIP prediction server that can keep these large memory footprint prediction models in memory while it is live. As a result, the predictions can be computed very fast through the web interface. A dataset containing 1000 peptides can be processed in seconds. Moreover, the user is no longer required to install and configure the MS^2^PIP tool, nor does the user require a machine with sufficient memory to run the models.

The prediction results are visualized online as MS^2^ spectra such that researchers can compare these predictions with the observations they make. For instance for manual post-validation of important peptide identifications such as peptide biomarkers or peptide transitions for targeted proteomics. The MS^2^ spectra can also be downloaded as spectrum files for archival or further processing. These predicted spectra can for instance be used to improve spectral library based peptide identification (4) or to select the best MS^2^ transitions (5) for a given protein in targeted proteomics.

## MS^2^PIP PREDICTION SERVER DESCRIPTION

### General framework

The MS^2^PIP prediction server is a python 2.7 Flask 0.10.1 web server (http://flask.pocoo.org/) that loads large python scikit-learn 0.15.1 (http://scikit-learn.org/stable/) Random Forest regression models and waits for peptides with desired MS^2^ spectrum charge states as input. The web interface is created using the Bootstrap 3 html framework (http://getbootstrap.com/) which enables the interface to adapt to different screen resolutions. For the visualization of the peak intensity predictions we used the D3.js javascript library (http://d3js.org/) combined with the C3.js reusable chart library (http://c3js.org/). Input data is sent straight to the prediction server and is destroyed immediately after predictions have been sent back to the user's browser where they can be downloaded and visualized.

### Input

In the first part of the input form the user has to provide the peptide sequence(s) with their desired MS^2^ spectrum charge states. You can use the tabs to either input one peptide directly or input up to a thousand peptides through file upload.

MS^2^PIP requires a peptide to be formatted as ‘H-P-OH’ where ‘H-’ is the amino terminal (N-terminal) molecule, ‘-OH’ is the carboxylterminal (C-terminal) molecule and ‘P’ is the amino acid peptide sequence. By default, both termini are assumed to be unmodified, which is represented by ‘H-’ for N-terminal hydrogen and ‘-OH’ for the C-terminal hydroxyl group. The peptide format ‘H-SAMPLE-OH’ is a valid input example.

Each amino acid residue in ‘P’ is represented by a capital letter, but it may be preceded by an arbitrary number of small letters to show a potential post-translational modification (PTM). For instance, ‘H-SAoxMPLE-OH’ contains a modification ‘ox’ for amino acid M at position 3 in the peptide sequence. The same applies for terminal modifications that replace the default ‘H-’ or ‘-OH’ symbols in the peptide format ‘H-P-OH’. For instance a terminal acetylation at the N-terminus is formatted as ‘ace-P-OH’. N-terminal and C-terminal modifications are also always written in lower case.

If the peptide contains PTMs the server needs to understand the meaning of the lower case PTM symbols used in the format of the peptide sequences. To make this process both simple and sufficiently general we adopted the python 2.7 pyteomics 2.5.5 library (http://pythonhosted.org/pyteomics/) that interfaces with the Unimod public domain database (http://www.unimod.org/). Unimod contains a large and detailed list of the most common PTMs and users can add their own PTMs to this public repository as well. We require the correct mass of the PTMs for the computation of the MS2PIP feature vectors and for the generation of the spectrum files. However, only few modifications listed in Unimod occurred in the training sets used to compute the prediction models. We listed the most frequent encountered PTMs used for training in Table 1. The user should be aware that the prediction models were trained to take modified amino acids into consideration, but not the specific type of modification. As a result the user should be very careful when using MS^2^PIP to investigate the effect of specific PTMs on the MS^2^ peak intensities. Further in this manuscript we will provide an evaluation for two commonly encountered artefactual PTMs (carbamidomethylcysteine and oxidized methionine) in an independent evaluation set.

**Table 1.** List of PTMs that occurred in more than 0.1% of the non-redundant peptides in the CID and HCD training sets

PTM	CID	HCD
Oxidation (M)	27.9	13.2
Label:13C(6) (n-term)	17.3	17.2
Propionyl (*)	13.4	8.0
Label:13C(6)+Acetyl (n-term)	11.0	<0.1
Propionyl (n-term)	9.8	6.9
Acetyl (n-term)	6.6	2.8
Carbamidomethyl (C)	5.7	1.7
Acetyl:2H(3) (n-term)	5.2	<0.1
Label:13C(6)15N(4) (n-term)	2.7	<0.1
Sulfide (M)	1.4	<0.1
Propionyl:13C(3) (K)	1.4	0.3
Deamidated (Q,N)	1.4	<0.1
Acetyl (*)	1.4	2.5
Butyryl (K)	0.5	0.4
Acetyl:2H(3) (*)	0.5	<0.1
Acetyl:13C(2) (n-term)	0.4	<0.1
Pro->Trp (C)	0.2	<0.1
Phospho (S,T,Y)	<0.1	0.9

The PTM names correspond to the PSI-MS name in Unimod.org, while the modified amino acids are presented between the brackets (‘(n-term)’ symbol is used to indicate that the PTM was observed as an amino-terminal modification. The numbers represent the percentage of non-redundant training PSMs that contained the modification.

In the second part of the input form the user has to link each lower case PTM symbol with the corresponding entry in Unimod. We provided a search box that lists all the PTMs contained in Unimod. For instance, for the server to understand that PTM ‘ox’ in ‘H-SAoxMPLE-OH’ means ‘Oxidation’ in the Unimod database the user needs to set the form element ‘ptm-symbol’ to ‘ox’ and select the PTM ‘Oxidation’ in the search box. N-terminal and C-terminal modifications are defined in the same way, but they end (N-terminal modification) or start (C-terminal modification) with the ‘-’ sign. For instance, to understand that N-terminal PTM ‘ace’ in ‘ace-SAMPLE-OH’ means ‘Acetylation’ in Unimod, the user needs to set the ‘ptm-symbol’ to ‘ace-’ and select the PTM ‘Acetylation’. Up to eight different PTMs are supported. The web server allows you to save your PTMs for future use.

The ‘MS^2^PIP’ button in the third part of the input form will submit the provided peptide sequences to MS^2^PIP to compute the MS^2^ peak intensity predictions.

### Output

Within seconds the prediction results are loaded in a new page. From here the user can download the CID and/or HCD MS^2^ peak intensity predictions as a ‘csv’ file or in the widely used Mascot Generic Format (MGF). The TITLE field for each predicted spectrum contains the input peptide sequence. The CHARGE field is set to the predicted spectrum charge state.

If the peptide contained PTMs then a list is provided with details about each modification as queried from Unimod. This allows the user to verify the masses found for each PTM. The user can download these PTMs for re-use during a next visit.

Finally we provide a visualization of the prediction(s) using the D3.js plotting framework. For each peptide we plot the mass peaks and the predicted intensities for the fragment ions b+, y+, b++ and y++. Peptides can be selected from a search box. Both CID and HCD predictions can be visualized in one plot for comparison.

## CID AND HCD PERFORMANCE

We showed that our prediction models compute CID MS^2^ spectra that correlate very well with the observations (3). We now show that the same approach works for HCD spectra as well. We trained HCD regression models on a set of 3 480 000 peptide-spectrum matches (PSMs) that were queried from the in-house ms-lims database (6). These PSMs were processed as described in (3) which resulted in 170 222 feature vectors for training the regression models for charge 2+ and 3+ HCD MS^2^ spectra.

We use an independent evaluation set that allows us to both evaluate the HCD prediction models and compare the results to those made by the CID prediction models that have been shown to be very accurate. This independent evaluation set is the result of analyzing a mixture of 4000 unique synthesized human peptides (7) that were predicted as proteotypic peptides using PeptideSieve (8) and then synthesized as peptide pools. All peptides consisted of 15 or 16 amino acids. Both CID (Orbitrap XL (35% normalized collision energy)) and HCD (Orbitrap Velos (30% normalized collision energy)) fragmentation spectra were generated using Mascot Distiller and represent de-isotoped and charge deconvoluted MS^2^ spectra. These files were searched against the human proteome using the Mascot (9) search engine 2.3.01. During the search we allowed for two common PTMs: carbamidomethylcysteine and oxidiation of methionine. For each PSM with *q*-value ≤ 1% we computed the Pearson correlation coefficient *R* between the predicted and the observed fragment ion peak intensities. Do note that the results on very different instrument types may be different.

**Figure 1.**
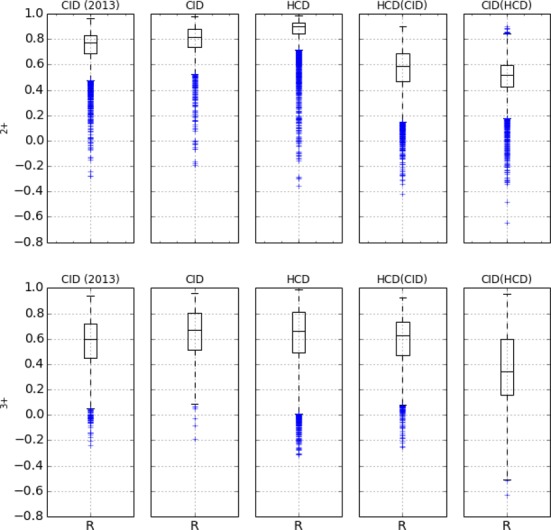
Grid of boxplots each representing the Pearson correlation (*R*) between predicted and observed MS^2^ peak intensities in the synthetic peptides dataset for the fragment ions listed in the text. Each box represents 50% of the data (Q1, Q2 and Q3), the lowest datum is at 1.5 × Q1 and the highest datum is at 1.5 × Q3. The top row contains the correlation distributions for the charge 2+ PSMs and the bottom row for the charge 3+ PSMs. The first column ‘CID (2013)’ shows the results for the original MS2PIP models. The second column ‘CID’ shows the results for the CID prediction models implemented in the prediction server. The third column ‘HCD’ shows the results for the HCD prediction models. The fourth column ‘HCD(CID)’ shows the results of applying the HCD prediction models on the CID dataset. The fifth column ‘CID(HCD)’ shows the results of applying the CID prediction models on the HCD dataset.

We first show that the new, modified Random Forest models in MS^2^PIP web server predict peak intensities with accuracy comparable to, or even better than, the original version of MS^2^PIP. Figure 1 (first two columns) plots the distribution of the *R*-values as boxplots for the original and the new prediction models. Indeed, the figure shows how the *R* distributions are very similar between both versions of MS^2^PIP for both charge states. For charge 2+ spectra the average correlation is 0.75 for the original MS^2^PIP and 0.78 for the modified MS^2^PIP applied by the server. For charge 3+ spectra, the accuracy of the models improved from 0.56 for the original to 0.64 for the modified MS^2^PIP. Additionally, the modified prediction models can compute MS^2^ spectra for all peptides without length restriction, whereas the original version was limited to lengths between 8 and 28.

The third column in Figure 1 shows the *R* distributions for the HCD prediction models. We observe that the predictions made by the HCD models are at least as good as those computed by the CID models. In fact, for charge 2+ we notice that the HCD models outperform the CID models, with average correlation equal to 0.78 for CID while this is 0.86 for HCD.

To justify the addition of separate HCD models we applied the CID prediction models on the identified HCD PSMs and computed the correlations *R* between these CID predictions and the HCD spectra observations. Similarly, we applied the HCD models on the identified CID PSMs and computed the correlation *R* between these HCD predictions and the CID spectra observations. So models trained for one fragmentation type are applied to predict the MS^2^ spectra for the other fragmentation type. The distribution of the *R* values in the last two columns of Figure 1 show a significant drop in prediction performance for both CID and HCD. This confirms the clear difference in fragmentation pattern between CID and HCD fragmentation, as already reported previously (10). This prompted the addition of the separate HCD models to the MS^2^PIP prediction server.

**Figure 2.**
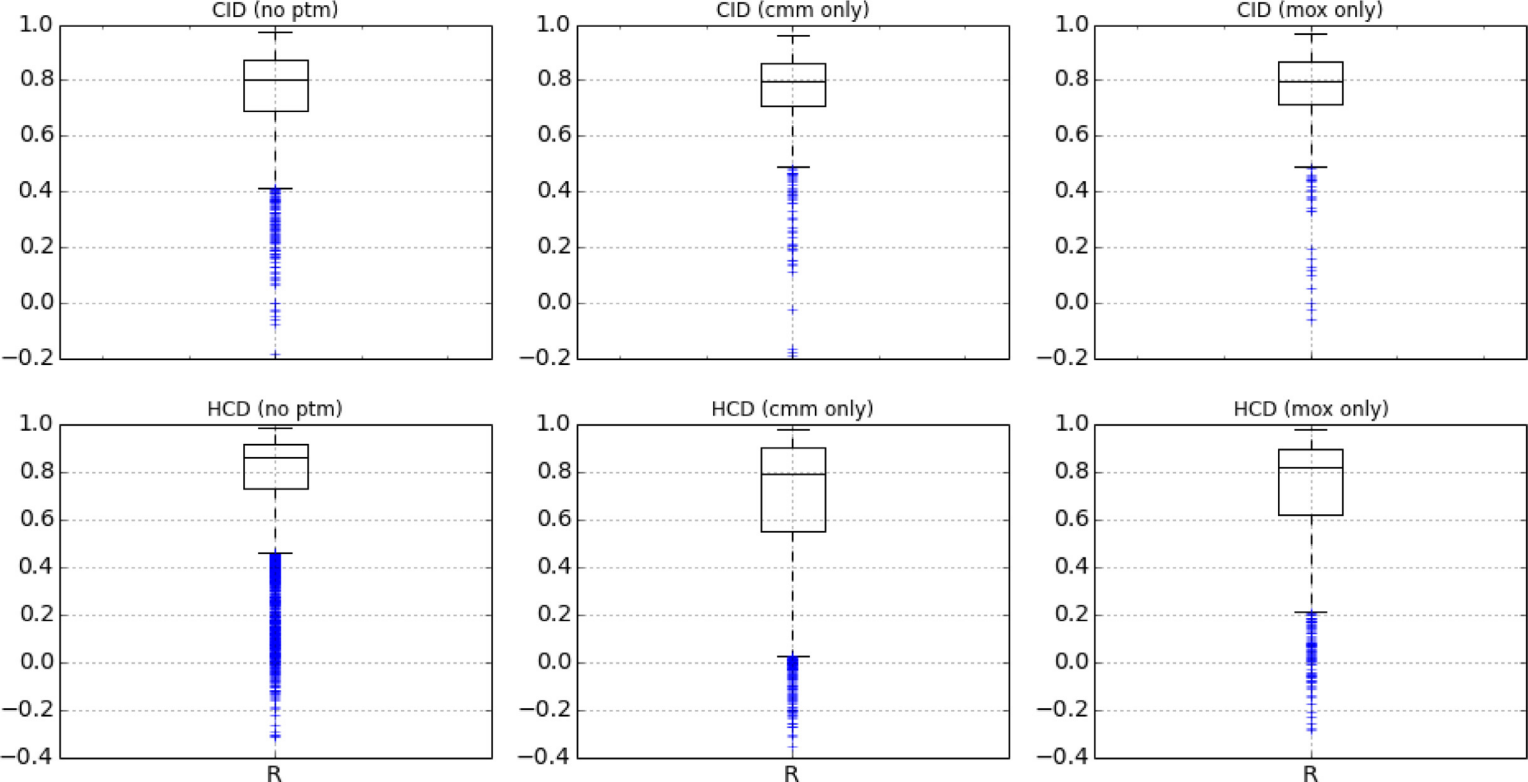
Grid of boxplots each representing the Pearson correlation (*R*) between predicted and observed MS^2^ peak intensities in the synthetic peptides dataset for the fragment ions listed in the text. Each box represents 50% of the data (Q1, Q2 and Q3), the lowest datum is at 1.5 × Q1 and the highest datum is at 1.5 × Q3. The top row contains the correlation distributions for the CID spectra, the bottom row for the HCD spectra. For each fragmentation type the results are partitioned based on the presence of a specific PTM in the identified peptide: Oxidation of methionine (mox) or Carbamidomethylcysteine (cmm). The first boxplots on each row show the results for the peptides that did not contain a PTM (no ptm).

In Figure 2 we partitioned the CID and HCD intensity correlation results based on the PTMs present in the identified peptides (carbamidomethylcysteine, oxidation of methionine or no PTM). For CID we observe no difference in median *R* values, the models perform well for both types of modifications. For HCD we also observe good performance for both types of PTMs, but in this case the *R* distribution of the unmodified peptides shows better performance. This could be due to the less frequent modification of amino acids observed in the HCD training set (Table 1).

## DISCUSSION

We here presented the online MS^2^PIP web server that enables users to create *in silico* predicted MS^2^ spectra based on input peptide sequences. MS^2^PIP server improves upon the original MS^2^PIP model and tool in three ways. First, the underlying prediction model has been changed to be more responsive and to remove the limitation on peptide lengths for which MS^2^ spectra can be predicted. Second, we have added a dedicated prediction model for HCD fragmentation in addition to the existing CID model. Third, the online prediction server obviates the need for local installation or user-side powerful compute facilities while providing a simple and efficient interface to run MS^2^PIP and visualize or download its output.
